# Mechanistic Insights and Real-World Evidence of Autologous Protein Solution (APS) in Clinical Use †

**DOI:** 10.3390/ijms26157577

**Published:** 2025-08-05

**Authors:** Jennifer Woodell-May, Kathleen Steckbeck, William King, Katie Miller, Bo Han, Vikas Vedi, Elizaveta Kon

**Affiliations:** 1 Zimmer Biomet, 56 E Bell Drive, Warsaw, IN 46580, USA; kathleen.steckbeck@zimmerbiomet.com (K.S.); katie.miller1@zimmerbiomet.com (K.M.); 2 Keck School of Medicine of USC, University of Southern California, 1333 San Pablo St., Los Angeles, CA 90033, USA; bohan@usc.edu; 3 Bishops Wood Hospital, Rickmansworth Rd, Northwood HA6 2JW, UK; vikas.vedi@hipkneespecialist.co.uk; 4 IRCCS Humanitas Research Hospital, Via Manzoni 56, 20089 Rozzano, Milan, Italy; elizaveta.kon@humanitas.it; 5 Department of Biomedical Sciences, Humanitas University, Via Rita Levi Montalcini 4, 20072 Pieve Emanuele, Milan, Italy

**Keywords:** M1 M2 macrophages, Osteoarthritis (OA), minimally clinically important improvement (MCII), Autologous protein solution (APS)

## Abstract

Autologous therapies are currently being studied to determine if they can modulate the course of knee osteoarthritis symptoms and/or disease progression. One potential therapeutic target is the polarization of pro-inflammatory M1 macrophages to pro-healing M2 macrophages. The autologous therapy, Autologous Protein Solution (APS), was incubated with donor-matched human peripheral-derived macrophages for 10 days. M1 pro-inflammatory macrophages were determined by the percentage of CD80+ and M2 pro-healing macrophages were determined by CD68+ and CD163+ by epifluorescent microscopy. To determine clinical effectiveness, an APS-specific minimal clinically important improvement (MCII) using an anchor-based method was calculated in a randomized controlled trial of APS (*n* = 46) and then applied to a real-world registry study (*n* = 78) to determine the percentage of pain responders. Compared to control media, APS statistically increased the percentage of M2 macrophages and decreased the percentage of M1 macrophages, while platelet-poor plasma had no effect on polarization. In the randomized controlled trial (RCT), the MCII at the 12-month follow-up visit was calculated as 2.0 points on the Western Ontario and McMaster Universities Osteoarthritis Index (WOMAC) pain scale and 7.5 points on the WOMAC function scale. Applying this MCII to the real-world registry data, 62.5% of patients met the MCII with an average of 4.7 ± 2.5 points of improvement in pain. Autologous therapies can influence macrophage polarization and have demonstrated clinical effectiveness in a real-world patient setting.

## 1. Introduction

Osteoarthritis (OA) is a debilitating disease driven by inflammatory cytokines and matrix metalloproteinases secreted by inflammatory M1 macrophages [1]. OA disease processes can result in impaired polarization of macrophages from M1 to M2 [2]. In an analysis of macrophages in synovial fluid from patients with knee OA, there were more M1 than M2 macrophages in the synovial fluid, supporting the hypothesis that M1 macrophages play a role in OA progression [3]. Furthermore, activated macrophages have been labeled with Etarfolatide in knee OA patients and the number correlated with OA severity [4]. Therefore, shifting the macrophage phenotype population from M1 to M2 could be a potential treatment target for knee OA progression.

Inflammatory (M1 phenotype) macrophages can be converted to a regenerative phenotype (M2 phenotype) in the presence of recombinant cytokines and growth factors such as interleukin-4 (IL-4), interleukin-13 (IL-13), interleukin-10 (IL-10), and transforming growth factor-β (TGF-β) [5]. As an alternative to an expensive single recombinant growth factor drug therapy, a white blood cell autologous therapy can be created at a patient’s point-of-care that will contain multiple cytokines and growth factors, including IL-4, IL-10, interleukin-1 receptor antagonist (IL-1ra), and TGF-β [6]. Buffy coat therapies (containing leukocytes and platelets) can support the transition from M1 to M2 dominant phenotypes and can resolve inflammation and enhance tissue repair [7]. Autologous point-of-care therapies concentrate cytokines in the picogram or nanogram per milliliter concentration as opposed to a milligram dose of a single recombinant therapy. Even so, autologous point-of-care therapies still provide an advantage of contributing a host of cytokines in a supraphysiological dose [8].

Assessment of both pain and functional improvement, coupled with determining whether the improvement is clinically meaningful for the patient, is necessary to evaluate the clinical efficacy of treatments for OA. The minimal clinically important improvement (MCII) is the smallest change in measurement that signifies a meaningful improvement in a patient’s condition. Instead of a general responder criterion defined by a fixed-point improvement in patient-reported outcomes (PROs), Tubach et al. defined a method whereby the specific MCII for a particular therapy is determined by anchoring pain and functional outcome scores to patient global impressions by asking the patients directly how they rate their own improvement [9]. Tubach’s method suggests that the MCII for an oral nonsteroidal anti-inflammatory drug (NSAID) may be different than the MCII for a specific intra-articular injection, and the methodology should be repeated for each individual therapy. Following the method outlined by Tubach, MCII was established for a novel intra-articular therapy in a randomized controlled trial and then applied to subjects enrolled in a real-world registry to determine the percentage of responders.

The aim of this study is to determine if APS can suppress the M1 macrophage phenotype, promote the M2 phenotype, and provide a minimal clinically important improvement in knee OA symptoms in real-world patients.

## 2. Results

### 2.1. M1 and M2 Macrophage Phenotype Assay

**Figure 1:**
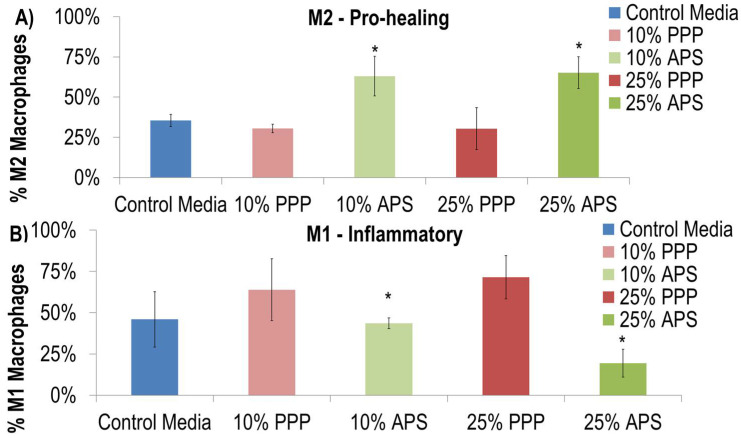
APS increased the differentiation of macrophages down the M2 lineage (**A**) (CD68+ and CD163+) and inhibited the differentiation of macrophages down the M1 pro-inflammatory lineage (**B**) (CD80+). * indicates *p* = 0.01; significant from control media after two-way ANOVA.

Autologous Protein Solution (APS) increased the percentage of M2 macrophages (CD68+ and CD163+) (Figure 1). Specifically, 35.5 ± 3.8% of macrophages maintained in control media had an M2 phenotype compared to 63.1 ± 12.4% and 65.2 ± 9.8% of macrophages maintained in 10% and 25% APS, respectively. Alternatively, the 10% and 25% doses of platelet-poor plasma (PPP) did not have a significant effect on the fraction of M2 macrophages (*p* > 0.05) (Figure 1A). APS reduced the percentage of M1 pro-inflammatory lineage cells (CD80+) in a dose-dependent manner. Specifically, 45.9 ± 16.7% of macrophages maintained in the control media had an M1 phenotype compared to 43.6 ± 3.2% and 19.4 ± 8.5% of macrophages maintained in 10% and 25% of APS, respectively (Figure 1B).

For the qualitative immunohistochemistry analysis, macrophages subjected to the STAT6 (signal transducer and activator of transcription 6) positive control condition appear to have the characteristics of an M2 polarized cell. The STAT6 positive control condition appeared to have nuclear staining with little to no STAT1 (signal transducer and activator of transcription 1) staining in the nucleus. In addition, there was no STAT6 staining in the cytoplasm. The STAT1 positive control had STAT1 nuclear staining, as well as some cytoplasmic staining. STAT1 staining remained primarily diffuse in the cytoplasm for all PPP, leukocyte-rich platelet-rich plasma (LR-PRP), and APS conditions, suggesting none of these autologous therapies resulted in a shift in polarization to M1 macrophages. STAT6 was localized in the nucleus in both the M2 positive-control media-treated cells and in the autologous therapy formulations. These results suggest that the white blood cells in the LR-PRP and APS do not adversely affect macrophage polarization.

### 2.2. Calculation of MCII by Anchor-Based Method (PROGRESS II)

**Figure 2:**
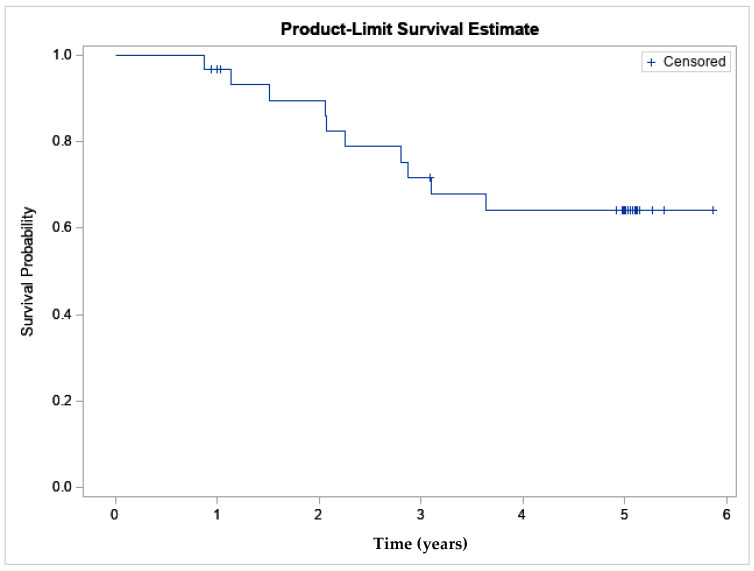
The Kaplan–Meier survivorship curve demonstrates survivorship of the APS cohort was 64% at 5 years (*n* = 31).

In order to determine if APS is clinically effective, data from a previously published clinical study were evaluated to calculate an MCII. A double blind, randomized, controlled trial (PROGRESS II) comparing the safety and efficacy of a single intra-articular injection of APS to saline, enrolling 46 subjects (31 APS, 15 saline) with a follow-up at 12 months, has previously been reported [10]. At 12 months, the percent improvement in Western Ontario and McMaster Universities Osteoarthritis Index (WOMAC) pain score was 65% in the APS group and 41% in the saline group (*p* = 0.02). At the final follow-up for the study (60 months), 17 of the original 31 randomized to APS remained in the study and had a mean WOMAC pain percent improvement of 59.7% (raw mean improvement: 6.8 ± 4.2), confidence interval [4.6, 8.9], and the average change from baseline was statistically significant (*p* < 0.0001). The Kaplan–Meier curve representing subjects exiting for knee-related events demonstrates a 0.64 survivorship probability [95% CI 0.463,0.820] at five years (Figure 2). This data suggests that while not all subjects are responders, there is a cohort that can achieve pain relief for 5 years without additional intra-articular or surgical treatment.

At the 12-month follow-up visit, MCII were derived using the anchor-based method with the Patients Global Impression of Improvement (PGI-I) and WOMAC pain and function outcomes. The mean improvement in WOMAC pain and function scores increased with each increasing patient rating (Table 1). The MCII was established as a 2.0-point improvement in WOMAC pain and a 7.5-point improvement in WOMAC function (Table 2).

### 2.3. Clinical Effectiveness in a Registry Study (PROGESS III)

The study population (% based on the number of joints) had 14.4% Kellgren Lawrence grade (KL) II, 65.4% KL III, and 20.2% KL IV. Knee Injury and Osteoarthritis Outcome Score (KOOS) pain improved from a baseline score of 49.7 ± 16.1 (*n* = 104 joints at baseline) to 64.0 ± 23.4 (*n* = 32 at 1 year) (14.3 points; 28.8% improvement; *p* = 0.0026 compared to baseline). KOOS pain and symptoms are reported per joint (Table 3), and KOOS daily living, sports and recreation, and quality of life (QoL) are reported per patient (Table 4). The numeric rating scale (NRS) pain scale improved 35% at one year compared to baseline scores (*p* ≤ 0.0001) (Table 3). Unilateral patients tended to score better on all PROs compared to bilateral patients. Six adverse events were reported in 4 patients, including excessive injection site pain or excessive injection site bruising. All adverse events were classified as mild (intervention not indicated), resolved, and were either possibly or definitely related to the procedure. As with most registries, the follow-up was a challenge. There was no follow-up incentive provided, and multiple subjects traveled significant distances to be treated. 37.2% (*n* = 29) of patients completed the 12-month time point. Thirty-three patients were lost to follow-up, two patients were withdrawn by the investigator for other reasons, 13 patients exited the study for another invasive OA treatment, and 1 patient voluntarily withdrew.

Using the specific APS calculated MCII, the percentage of pain responders was determined from the PROGRESS II and PROGRESS III clinical studies (Table 5). All the PROGRESS II APS cohort subjects that completed the 60-month time point were 12-month pain responders. For the PROGRESS III subjects, the KOOSs were converted to WOMAC in order to apply the WOMAC MCII. A total of 62.5% of subjects that met the MCII had an average 12-month WOMAC pain score of 3.7 ± 2.5, and the 37.5% that fell below the MCII had a 12-month WOMAC pain score of 10.1 ± 4.5.

## 3. Discussion

Autologous therapies may provide cytokine profiles that can ameliorate symptoms and potentially modulate disease progression. As autologous therapy output differs based on the preparation method, there has been significant interest in comparing the various preparation techniques and outputs, both in cell content and resulting cytokine concentrations. APS has a higher concentration of WBC than traditional LR-PRP (46 k/µL versus 28 k/µL WBC) and lower platelet concentration (707 k/µL versus 1745 k/µL PLT). Both APS and LR-PRP are more similar than leukocyte-poor platelet-rich plasma (LP-PRP) (1.5 k/µL WBC and 399 k/µL PLT) [11]. Additionally, Wakayama et al. compared the cellular and cytokine outputs of APS and LP-PRP and found significantly higher concentrations of platelets, leukocytes, interleukin 1 receptor antagonist (IL-1Ra), soluble tumor necrosis factor receptor type II (sTNF-RII), interleukin 1β (IL-1β), and the ratio of IL-1Ra/IL-1 β in APS, whereas the platelet derived growth factor subunit BB (PDGF-BB) was higher in LP-PRP [12].

In this study, APS significantly modulated a shift in M1 and M2 macrophage polarization while PPP did not. In the immunohistochemical analysis, neither PPP, LR-PRP, nor APS shifted macrophages to the M1 phenotype, and all autologous therapies shifted macrophages towards M2. These results suggest that autologous therapies that are inclusive of white blood cells do not negatively influence macrophage polarization. Uchiyama et al. [13] compared humeral factors from LP-PRP and APS and found that APS had higher concentrations of the M2-related factors IL-10 and TGF-β. As demonstrated in this study, culture with monocyte-derived macrophages demonstrated that both formulations reduced the expression of M1 macrophage markers and promoted polarization from M1 macrophages to M2 macrophages. M2 macrophages can be subdivided into two types, including M2a, relating to anti-inflammatory activity, and M2c, relating to tissue repair. Uchiyama et al.’s results further elucidate the distinction between types of M2 macrophages by demonstrating that LP-PRP promoted a shift in polarization to M2c tissue repair type and APS promoted a shift in polarization to M2a anti-inflammatory type. When considering the inflammatory nature of OA, the various M2 phenotypes could potentially alter the M1/M2 balance differently within the osteoarthritis joint [13]. Additionally, comparing the cell phenotypes with flow cytometry, both pre- and post-APS processing, the monocyte population was enriched in the M2-like dominant phenotype [14]. Similarly, in a mouse tendon injury model, both LP-PRP and LR-PRP improved tendon healing compared to controls, but differences were observed in angiogenesis (>LR-PRP) versus collagen arrangement (>LP-PRP) and the time course of M1 and M2 phenotypes and M1/M2 ratios [15].

A significant amount of discussion regarding the safety and efficacy of autologous therapies for treatment of OA has focused on the role of white blood cells as they can produce both inflammatory (IL-1 and tumor necrosis factor-α (TNF-α)) and anti-inflammatory (IL-1ra, sTNFRs, and soluble interleukin-1 receptors (sIL1Rs)) factors. In a matched-donor comparison of LR-PRP and LP-PRP in patients with mild to moderate knee OA, LR-PRP expressed significantly more IL-1ra, IL-4, IL-8, and matrix metalloproteinase-9 (MMP-9) compared to LP-PRP [16]. Mariani et al. injected LR-PRP or hyaluronic acid into subjects with knee OA and compared synovial fluid aspiration for inflammatory markers IL-1 and IL-6 at 2, 6, and 12 months post-injection and found no significant differences between treatments. They conclude that LR-PRP does not create an up regulation of inflammatory cytokines when treating knee OA [17]. Romandini et al. conducted a double-blind, randomized, controlled trial comparing the LP- and LR-PRP, and at follow-up out to 12 months, did not find statistical differences in safety and efficacy outcomes [18]. In the European Society of Sports Traumatology, Knee Surgery, and Arthroscopy Orthobiologics Initiative (ESSKA-ORBIT) consensus paper, leading experts evaluating the current literature concluded there is enough preclinical and clinical evidence to support the use of PRP for treating knee OA (Grade A) and no significant differences between LR-PRP and LP-PRP clinical outcomes (Grade B) [19].

Further supporting the use of leukocyte-rich anti-inflammatory products, the inflammatory and anti-inflammatory cytokines in APS and LR-PRP are predominantly produced by the white blood cells. LP-PRP has significantly fewer white blood cells (~1.5 k/µL), IL-1 (below detection limit), and IL-1ra (~673 pg/mL) compared to APS, which contains more white blood cells (~46.5 k/µL), IL-1 (~3.8 pg/mL), and IL-1ra (~30,853 pg/mL) [11]. Similar cytokine profiles have also been observed in equine autologous anti-inflammatory products [20]. In APS, the white blood cell concentration significantly correlates to the concentration of IL-1ra but not IL-1. In this same study evaluating pain in the treatment of knee OA, Outcome Measures in Rheumatology—Osteoarthritis Research Society International (OMERACT-OARSI), high pain responders had an IL-1ra: IL-1 ratio greater than 1000 [21]. A recent systematic review has identified that platelet dose may correlate to clinical outcomes in OA at 12 months [22]. Together, both clinical and laboratory research suggest APS is an optimized autologous anti-inflammatory product for addressing OA.

There are several methodologies to determine whether a treatment is clinically effective for knee OA. In this study, the anchor-based method developed by Tubach et al. was utilized to develop a minimal clinically important difference for the subjects by anchoring their change in pain score to their own evaluation of their perceived level of improvement [9]. It is common to utilize a 10-point change in KOOS pain as clinically significant [23]. However, when comparing these two methods, the 10 points on the KOOS pain method found 84.4% of subjects as responders in the real-world evidence trial (PROGRESS III), while the anchor-based method found only 62.5% of subjects as responders. While the MCII calculated in this study and the KOOS MCII both represent 10% of their respective pain scales, the anchor-based method was more conservative in defining responders. The anchor method may provide more specificity to the actual treatment modality compared to the evaluation of a standard point change across a patient-reported outcome.

In comparison, the anchor-based method for the randomized, controlled trial (PROGRESS II) found 80.4% of subjects as MCII responders. This discrepancy with PROGRESS III highlights one of the major differences between RCTs and registries. In RCTs, often the inclusion and exclusion criteria of the study are designed to tightly control the patients enrolled in the study to best detect safety and efficacy signals. These study designs are often required by regulatory agencies to approve new therapies. However, RCTs with tight enrollment criteria do not always reflect how a therapy will perform when treating all treatable patients. For example, in the RCT PROGRESS II, subjects with KL2/3, a minimum and maximum allowable initial pain score, and only unilateral OA were enrolled. None of these restrictions were required in the registry PROGRESS III, allowing any amount of pain, KL grade, or unilateral or bilateral OA. This is therefore reflected in the number of responders in each study design. Similarly, Kuwasawa et al. described real-world patients (n=220 knees) diagnosed with varying severities of OA treated with APS and followed for 12 months with a responder rate of 80% for KL2, 73% for KL3, and 45% for KL 4 [24]. Both study designs can provide useful information on which subjects are best suited for APS treatment.

While knee OA symptom relief is the primary goal for treatment with autologous therapies, there is hope that these treatments could alter the course of disease progression. While no study has demonstrated these treatments as true disease-modifying osteoarthritis drugs (DMOADs) to date, there are encouraging animal and human clinical studies where instances of improved cartilage thickness have emerged. In animal models of OA, APS has improved gross histopathology scores compared to controls for IL-1-induced synovitis [25] and decreased cartilage degradation compared to controls in a meniscal injury model [26]. In human clinical trials, Sekiya et al. measured some subjects with increased cartilage thickness in the medial knee OA six months after treatment with APS [27]. While not statistically significant, there were also subjects treated with APS in the PROGRESS II trial that demonstrated increased cartilage thickness at the 12-month follow-up visit. These results from Sekiya et al. [27] and PROGRESS II are early exploratory findings and need to be repeated in larger studies to confirm the results.

It appears that polarization of macrophages is a potential therapeutic target for both OA symptom relief and potentially to alter disease progression. Autologous therapies can contain a multitude of cytokines that can push M1 macrophages into an M2 phenotype. Determination of clinical effectiveness both in controlled trials and real-world registries can provide meaningful information to help determine which patient candidates are best suited for this type of treatment. Recommended future work would be repeating the clinical studies with a higher number of subjects and with a direct correlation of M1 and M2 phenotypes to compare the long-term responder and non-responder analysis to the macrophage profile.

## 4. Materials and Methods

### 4.1. Macrophase In Vitro Analysis

The blood from four human donors was processed using the APS device system (nSTRIDE^®^ APS Kit, Zimmer Biomet, Warsaw, IN, USA) to produce APS and PPP. Informed consent was obtained from all subjects involved in the study (WIRB 1-700813-1; protocol HBD-001). APS was processed from 60 mL of anticoagulated whole blood in approximately 20 minutes. Peripheral blood monocytes were acquired from the same donors using Ficoll separation techniques (Stem Cell Technologies, Vancouver, BC, Canada) and were subsequently cultured using peripheral blood cell macrophage control media (Promocell, Heidelberg, Germany) or control media with either 10% PPP, 25%PPP, 10% APS, or 25% APS. Cells were cultured for 10 days and analyzed using epifluorescent microscopy. M1 pro-inflammatory macrophages were determined by the percentage of CD80+ (M1 profile marker) and M2 pro-healing macrophages were determined by CD68+ (pan macrophage marker) and CD163+ (M2 profile marker).

In addition, blood from 4 additional human donors was collected and processed to create donor-matched PPP, LR-PRP, and peripheral monocytes. The LR-PRP device concentrates platelets 9-fold over baseline, and WBC 5-fold over baseline, and the APS concentrates platelets 4-fold over baseline and WBC 8.6-fold over baseline whole blood. The peripheral monocytes were cultured with macrophage attachment medium (Promocell, PN: C-28052, Heidelberg, Germany) and then in macrophage attachment medium (Promocell, PN: C-28057, Heidelberg, Germany) for 6 days. After 6 days in culture, cells were incubated with 50 ng/mL interferon Υ (IFN-Υ) (Abcam Inc., PN: ab9659, Cambridge, MA, USA) as MI positive control media, 20 ng/mL IL-4 (Abcam Inc., PN: ab73186, Cambridge, MA) as M2 positive media, or 10% and 25% of PPP, LR-PRP, and APS, respectively (*n* = 3 per donor). On day 10, cells were processed for qualitative immunohistochemistry staining. Wells were incubated with either STAT1 primary antibody (Abcam Inc., PN: ab2415, Cambridge, MA) or STAT6 primary antibody (Santa Cruz Biotech, PN: sc-374021, Dallas, TX, USA), followed by a secondary antibody and then DAPI (4′,6-diamidino-2-phenylindole) mounting solution. Trends in localization of STAT1 in the cell nucleus indicate classically activated macrophages that are a pro-inflammatory phenotype (M1), while STAT6, predominantly located in the nucleus, indicates macrophages have been alternatively activated and have a pro-healing phenotype (M2).

### 4.2. Calculation of Minimally Clinically Important Improvement (MCII)

A randomized, double blind, placebo-controlled clinical trial (PROGRESS II: NCT02138890) was utilized to evaluate the effect of a single injection of APS in subjects with mild to moderate knee OA [10]. The clinical study was approved by ethics committees, and all subjects signed informed consents before enrollment. Subjects with KL 2 or 3 unilateral knee OA were included in the study. The APS cohort was asked to participate in an unblinded 60-month follow-up, and the saline patients were offered cross-over after 12 months and were similarly followed. Efficacy endpoints WOMAC LK 3.1 (The Western Ontario and McMaster Universities Osteoarthritis Index Likert scale 3.1) and VAS (Visual Analog Scale) were measured as a change from baseline to each time point.

Subjects were asked at the 12-month follow-up visit to rate their improvement using a Patient Global Impression of Improvement scale (PGI-I) (“Very Much Improved”, “Improved”, “Minimally Improved”, “No Change”, “Minimally Worse”, “Much Worse”, and “Very Much Worse”). WOMAC (LK3.1) pain (0–20) and function (0–68) score changes from baseline to 12 months were then calculated for subjects scoring themselves as “minimally improved”. The MCII was established from the 25th percentile distribution of WOMAC pain and function scores (improvement from baseline) from all randomized subjects. The 25th percentile of the distribution of WOMAC pain scores (improvement from baseline) for these subjects corresponds to a score achieved by 75% of the patients reporting a “minimally improved” outcome [28].

### 4.3. Post-Market Registry Data Collection

Seventy-eight patients (*n* = 104 joints) were enrolled in a prospective, longitudinal case series design in a single center in the United Kingdom (PROGRESS III, NCT02580643). Informed consent was obtained from all subjects involved in the study. Unlike in the previous RCT, inclusion criteria included either unilateral or bilateral knee OA, and the patient′s age was at least 18 years old. There were no limitations of either radiologic grade of OA (Kellgren and Lawrence scale, KL) or initial presenting pain level. Outcome measures included KOOS, NRS (pain, stiffness, and function), EuroQol 5-Dimension (EQ-5D), and adverse events and complications were collected for 12 months. In order to apply the WOMAC pain-derived MCII, the WOMAC score was extracted from the KOOS, and the MCII was applied to the WOMAC score [23].

### 4.4. Statistical Analysis

Data is presented as mean ± standard deviation. Statistical significance was determined with statistical software (Minitab, Inc., State College, PA, USA). Normality was analyzed using the Anderson—Darling test (α = 0.05). A two-way ANOVA was performed on the epifluorescent microscopy data (α = 0.05). If both preoperative and postoperative values were normally distributed in the clinical studies, a paired t-test was performed (α = 0.05). If variables were not normally distributed, then a Wilcoxon Sign Rank test was performed (α = 0.05). Clinical patient-reported outcomes data were calculated on the per-protocol data set.

## Figures and Tables

**Table 1:** WOMAC pain and function scores per category of the transition PGI-I question. Point improvement for WOMAC pain and function was calculated from baseline to the 12-month follow-up visit.

Transition Question	*n*	WOMAC Pain Improvement	WOMAC Function Improvement
Very Much Worse	1	−1.0	−7.0
Much Worse	1	1.5	4.5
Minimally Worse	5	3.2	3.8
No Change	6	4.2	7.4
Minimally Improved	9	5.8	15.8
Much Improved	18	8.4	25.3
Very Much Improved	4	10.9	37.8

**Table 2:** MCII point improvement from baseline to 12 months.

Transition Question	MCII for WOMAC Pain Improvement	MCII for WOMAC Function Improvement
Based on the 25th percentile of the PGI—“Minimally Improved” subjects	2.0	7.5

**Table 3:** KOOS pain and symptom subscales and NRS outcomes. Data presented as mean (SD). *p* value is comparing 12 months to the baseline score.

PRO (*n* = Joints)		Baseline (*n* = 104)	2 Weeks (*n* = 57)	4 Weeks (*n* = 62)	3 Months (*n* = 51)	6 Months (*n* = 40)	12 Months (*n* = 32)	*p* Value
KOOS	Pain	49.7 (16.1)	60.1 (18.9)	60.2 (18.8)	63.4 (21.6)	57.9 (25.2)	64.0 (23.4)	*p* < 0.01
	Symptoms	53.6 (18.6)	62.7 (16.7)	63.0 (16.7)	62.6 (20.4)	57.3 (23.7)	62.1 (23.8)	*p* < 0.01
NRS	Pain	5.8 (2.3)	4.3 (2.5)	4.3 (2.5)	4.0 (2.6)	4.7 (2.7)	3.7 (2.8)	*p* < 0.01
	Function	5.1 (2.2)	3.8 (2.6)	4.2 (2.6)	4.1 (2.5)	4.3 (2.5)	3.5 (2.6)	*p* < 0.01
	Stiffness	5.3 (2.5)	4.0 (2.3)	4.0 (2.3)	3.6 (2.2)	4.2 (2.6)	3.7 (2.9)	*p* < 0.01

**Table 4:** KOOS activities of daily living, sport and recreation function, and quality of life (QoL) subscale outcomes. Data presented as mean (SD). *p* value is comparing 12 months to the baseline score.

PRO (*n* = Subjects)		Baseline (*n* = 78)	2 Weeks (*n* = 44)	4 Weeks (*n* = 48)	3 Months (*n* = 40)	6 Months (*n* = 32)	12 Months (*n* = 26)	*p* Value
KOOS	Daily Living	62.9 (21.6)	64.8 (21.9)	67.6 (20.9)	61.3 (25.3)	61.5 (22.7)	64.6 (25.7)	*p* = 0.537
	Sports and Rec	32.8 (25.9)	30.2 (24.8)	37.3 (29.1)	36.6 (30.5)	37.7 (27.3)	36.0 (29.6)	*p* = 0.524
	QoL	34.8 (23.2)	36.8 (23.4)	41.3 (25.3)	34.3 (21.9)	42.1 (25.4)	40.2 (26.1)	*p* = 0.649

**Table 5:** MCII responds to pain.

Study	Time Point	Category	Pain Responders % (*n*)	Average Pain Point Improvement Mean (SD)
PROGRESS II RCT	12 mo.	Meets MCII	80.4% (37/46)	7.5 (3.0)
		Below MCII	19.6% (9/46)	−0.78 (2.2)
	60 mo.	Meets MCII	88.2% (15/17)	7.8 (3.2)
		Below MCII	11.7% (2/17)	−1.0 (2.8)
PROGRESS III Registry	12 mo.	Meets MCII	62.5% (20/32)	4.7 (2.5)
		Below MCII	37.5% (12/32)	−1.25 (2.3)

## Data Availability

The data presented in this study are only available on request from the corresponding author due to privacy reasons.

## Abbreviations

The following abbreviations are used in this manuscript:

DMOAD	Disease modifying osteoarthritis drug
IL-1	Interleukin-1
IL-1ra	Interleukin-1 receptor antagonist
IL-4	Interleukin-4
IL-10	Interleukin-10
LP-PRP	Leukocyte poor platelet-rich plasma
LR-PRP	Leukocyte rich platelet-rich plasma
KOOS	Knee Injury and Osteoarthritis Outcome Score
MCII	Minimal Clinically Important Improvement
NRS	Numeric rating scale
OA	Osteoarthritis
PGI-I	Patient Global Impression of Improvement Scale
PPP	Platelet-poor plasma
PRO	Patient reported outcomes
TGFβ	Transforming growth factor-beta
TNFα	Tissue necrosis factor-alpha
sIL-1R	Soluble Interleukin-1 receptor
sTNFR	Soluble Tissue necrosis factor receptor
VAS	Visual Analog Scale
WOMAC	The Western Ontario and McMaster Universities Osteoarthritis Index
